# Practical Approaches to Transvenous Lead Extraction Procedures—Clinical Case Series

**DOI:** 10.3390/ijerph20010379

**Published:** 2022-12-26

**Authors:** Paul-Mihai Boarescu, Iulia Diana Popa, Cătălin Aurelian Trifan, Adela Nicoleta Roşian, Ştefan Horia Roşian

**Affiliations:** 1 Department of Pharmacology, Toxicology and Clinical Pharmacology, Iuliu Haţieganu University of Medicine and Pharmacy Cluj-Napoca, Gheorghe Marinescu Street, No. 23, 400337 Cluj-Napoca, Romania; 2 “Niculae Stăncioiu” Heart Institute Cluj-Napoca, Calea Moților Street, No. 19-21, 400001 Cluj-Napoca, Romania; 3 Department of Cardiovascular Surgery, “Iuliu Haţieganu” University of Medicine and Pharmacy Cluj-Napoca, 19-21 Calea Moților Street, 400001 Cluj-Napoca, Romania; 4 Department of Cardiology—Heart Institute, “Iuliu Haţieganu” University of Medicine and Pharmacy Cluj-Napoca, 19-21 Calea Moților Street, 400001 Cluj-Napoca, Romania

**Keywords:** transvenous lead extraction, cardiac implantable electronic devices, clinical cases

## Abstract

Transvenous lead extraction (TLE) is regarded as the first-line strategy for the management of complications associated with cardiac implantable electronic devices (CIEDs), when lead removal is mandatory. The decision to perform a lead extraction should take into consideration not only the strength of the clinical indication for the procedure but also many other factors such as risks versus benefits, extractor and team experience, and even patient preference. TLE is a procedure with a possible high risk of complications. In this paper, we present three clinical cases of patients who presented different indications of TLE and explain how the procedures were successfully performed. In the first clinical case, TLE was necessary because of device extravasation and suspicion of CIED pocket infection. In the second clinical case, TLE was necessary because occlusion of the left subclavian vein was found when an upgrade to cardiac resynchronization therapy was performed. In the last clinical case, TLE was necessary in order to remove magnetic resonance (MR) non-conditional leads, so the patient could undergo an MRI examination for the management of a brain tumor.

## 1. Introduction

As more people have been living longer in recent decades, the use of cardiac implantable electronic devices (CIEDs) has increased significantly. CIEDs include permanent pacemakers (PPMs) and implantable cardioverter-defibrillators (ICDs). Since the beginning of the 21st century, there has been an expansion of the indications for CIEDs. Even more, device therapy has become more complex, frequently involving multiple leads per patient and more sophisticated devices [1].

Lead removal is currently a specialized procedure with well-defined indications, as patients’ longer life expectancy has led to an increase in the number of device-related complications and, consequently, to an increased need to perform lead-removal procedures [2]. 

Transvenous lead extraction (TLE) is regarded as the first-line strategy for the management of complications associated with CIEDs when lead removal is mandatory [3]. Minor complications of TLE, which include bleeding, pocket hematoma, pneumothorax, venous thrombosis, and migrated lead fragments, even if they are usually not life-threatening, are significant and require rapid intervention [4]. Pericardial tamponade is the most common major complication. Other major complications include hemothorax, thromboembolic events, vascular laceration, cardiac avulsion, and even death. Some of the considerable TLE complications may entail emergent sternotomy and surgical repair, and, in rare cases of rapid and massive blood loss, death is often the outcome [4]. The rate of the major complications that require emergent intervention were reported to be low (up to 2.2% of cases) [5,6], while the mortality rate was reported to be exceedingly low (0.3%) [7]. Male gender, age during TLE, higher NYHA class, low left ventricle ejection fraction, presence of atrial fibrillation, and chronic renal failure were identified as the main factors predicting shorter survival among patients undergoing TLE [8].

The present study aims to highlight the indications and the methods of transvenous lead extraction and to present some clinical cases where lead extraction was mandatory and procedures were successfully performed without major or minor complications.

## 2. Clinical Cases

All the procedures were performed in the electrophysiology room, with patients in deep analgesia-sedation and monitored by an anesthesiologist. Moreover, a cardiothoracic surgeon and a cardiovascular surgery operating room were on stand-by in the same building.

### 2.1. First Clinical Case

#### 2.1.1. Patient’s Medical History and Presentation

**Figure 1:**
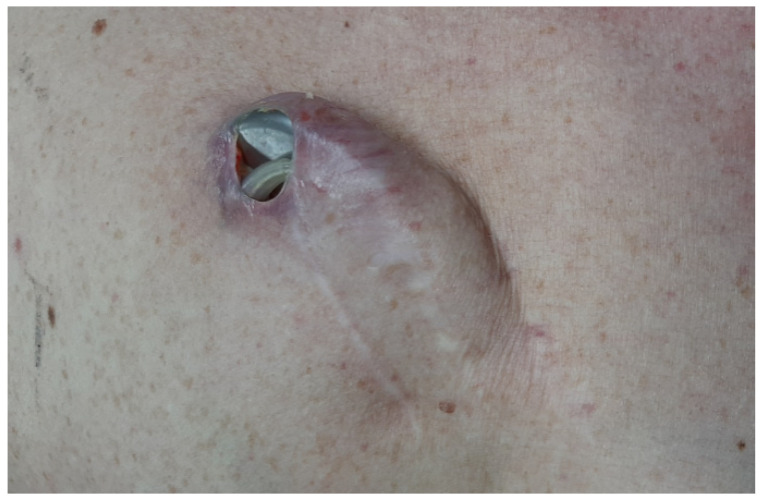
The aspect of the pocket erosion with the extravasation of the device and the lead, at the moment of admission to the hospital.

A 54-year-old male with a history of a chronically implanted ICD (at another center in a foreign country) was initially admitted to the Cardiology Department because of pocket erosion and possible pocket infection, as shown in Figure 1.

The medical history of the patient revealed that he has three-vessel coronary artery disease and that his first ICD, with a passive fixation double-coil lead, was implanted 11 years before this admission, as the primary prevention of sudden cardiac death. After three years, due to inappropriate internal electric shocks, a diagnosis of lead fracture was established. A new dual-coil active fixation lead was implanted, and the ICD generator was replaced without extraction of the old lead. Due to battery discharge, the ICD generator was replaced again one year later.

**Figure 2:**
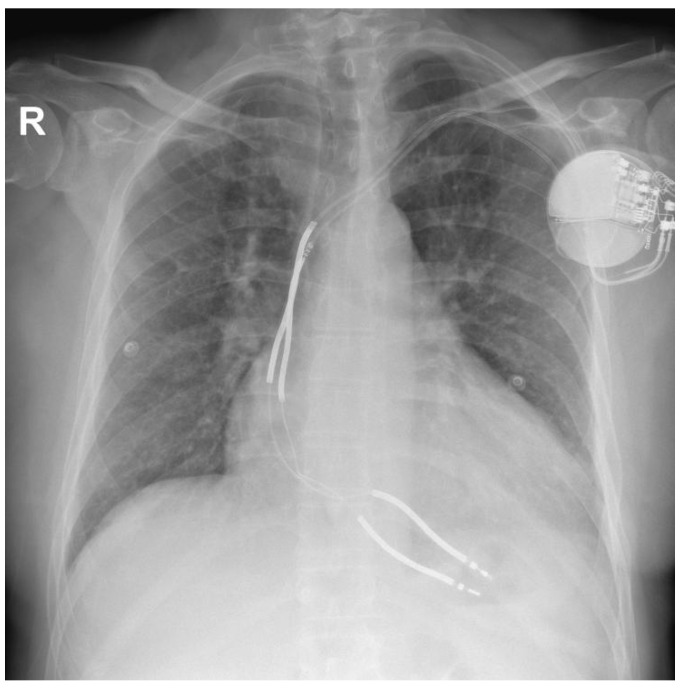
Chest X-ray at the time of admission at the hospital.

At the time of admission to our department, the patient was asymptomatic. The ECG showed a sinus rhythm with left bundle-branch block. A chest X-ray revealed an increased cardiothoracic ratio, a pulse-generator ICD placed subcutaneously in the left subclavian area, and two dual-coil defibrillation leads in the apex of the right ventricle (Figure 2).

The transthoracic echocardiogram revealed a markedly dilated left ventricle, severe reduction in global contractility (EF: 21%), a restrictive filling pattern, moderate mitral regurgitation, and moderate tricuspid regurgitation without visualization of vegetations. A transesophageal echocardiography was also performed, and no intracavitary vegetation was identified at the tricuspid valve or leads’ level.

Biochemical evaluation excluded systemic inflammation, as C reactive protein, fibrinogen, and the erythrocytes sedimentation rate were all in normal ranges. The blood cultures and wound-drainage cultures were also negative.

#### 2.1.2. Transvenous Leads Extraction Procedure

The ICD pulse generator was interrogated using a corresponding programmer, which revealed repeated episodes of fast ventricular tachycardia (VT), interpreted as ventricular fibrillation (VF), with adequate internal electric shock administration. The last episode was recorded 10 months ago. Before starting the procedure, the anti-tachycardia therapies were discontinued. CIED fluoroscopy was performed to identify the position of the ICD pulse generator in the left subclavian area. One double coil, with an active fixation ICD lead (dwell time 8 years), was connected to the pulse generator and tracked normally. Another double coil, with a passive fixation ICD lead (dwell time 11 years), was noted to be fractured, with the proximal end lead remnant in the ICD pocket. Iodinated contrast media was injected into the peripheral vein of the left arm, without revealing subclavian or brachiocephalic vein occlusion.

After deep analgesia-sedation, an incision was made, and the pulse generator was removed from the pocket. Afterward, the leads were dissected to the level of the anchoring sleeves, to free them up from the scar tissue. The lead connected to the ICD pulse generator was disconnected, the tie-down sutures were removed, and gentle traction was initiated to remove the lead, without success. The connector of the lead was cut off and a Liberator locking stylet was advanced and locked at the tip of the lead. The Liberator locking stylet was secured on the lead using a one-tie accessory. With continuous traction, a 13 French bidirectional, rotational, mechanical lead-extraction sheath (Evolution RL, Cook Medical, USA) was advanced over the lead, up to its tip, to break up the heavy fibrosis from the left innominate vein, lateral wall of the left atrium, proximal pole of the lead, and lead’s tip. After removing the fibrosis, continuous gentle traction dislodged the lead, and it was completely removed without difficulty (as shown in Supplementary Video S1). 

A similar technique was used for the abandoned lead, and the same 13 French bidirectional rotational mechanical lead extraction sheath was used and advanced over the lead, up to its tip, to break up the heavy fibrosis near the superior vena cava, lateral wall of the left atrium, tricuspid valve, the proximal pole of the lead, and lead’s tip. After removing the heavy fibrosis, continuous gentle traction dislodged the lead, and it was completely removed without incident (as shown in Supplementary Video S2).

The overall fluoroscopy time for this procedure was 4 min and 13 s.

**Figure 3:**
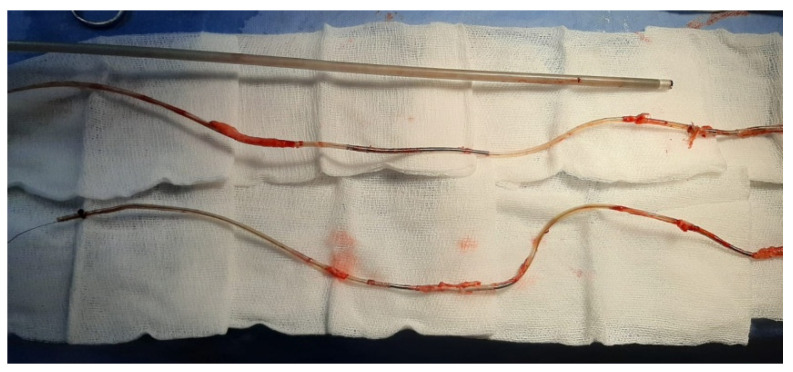
Extracted leads with extensive fibrosis.

The extracted leads with extensive fibrosis, especially on the coils of the leads, are shown in Figure 3.

A transthoracic echocardiogram was performed immediately after the procedure in the electrophysiology room. No pericardial effusion tricuspid valve dysfunction, or lead fragments were found. Subsequently, the patient was monitored (also by echocardiography) for 24 h in the intensive care unit for critical cardiac patients.

**Figure 4:**
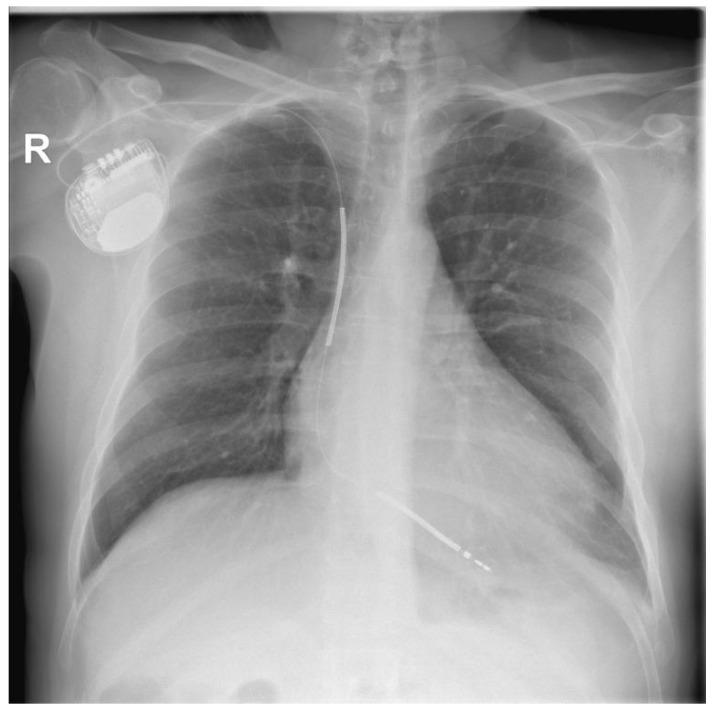
Chest X-ray with new defibrillator on the right side of the chest and the lead implanted through the right subclavian vein.

After 16 days of antibiotic therapy with Vancomycin, a new ICD was implanted on the right side of the chest (Figure 4). 

### 2.2. Second Clinical Case

#### 2.2.1. Patient’s Medical History and Presentation

A 67-year-old male patient with a dual-chamber pacemaker was admitted to the Cardiology Department for an upgrade to his cardiac resynchronization therapy due to impaired systolic function. The patient was diagnosed with ischemic cardiomyopathy, chronic heart failure (HF) with III New York Heart Association (NYHA) functional status, and a dual-chamber pacemaker for third-degree atrioventricular block had been implanted seven years before the current admission to the hospital.

At the time of admission to our department, the patient complained of considerable limitations to physical activity due to shortness of breath and fatigue despite optimal medical therapy. An ECG showed an atrial sinus rhythm with a ventricular-paced rhythm. A chest X-ray revealed an increased cardiothoracic ratio, a pulse generator PM placed subcutaneously in the left subclavian area, and two bipolar active fixation leads—one in the right atrial appendage and the other on the apex of the right ventricle.

The echocardiogram revealed a mildly dilated left ventricle, a severe reduction in global contractility (EF: 23%), a restrictive filling pattern, mild aortic regurgitation, moderate mitral regurgitation, moderate tricuspid regurgitation, diffuse hypokinesia, and no visible vegetations.

A pacemaker interrogation revealed 100% atrial sensed, 100% ventricular paced, and no underlying rhythm, with both leads having impedance, pacing, and sensing parameters within normal limits.

#### 2.2.2. Transvenous Leads Extraction Procedure

The first intention was to implant only the left ventricular lead (LV) in a branch of the coronary sinus (CS), maintaining the pre-existing RA and RV leads for an upgrade to a CRT-P, although, according to current guidelines [9], the indication was that a CRT-D would provide the patient maximum benefits.

**Figure 5:**
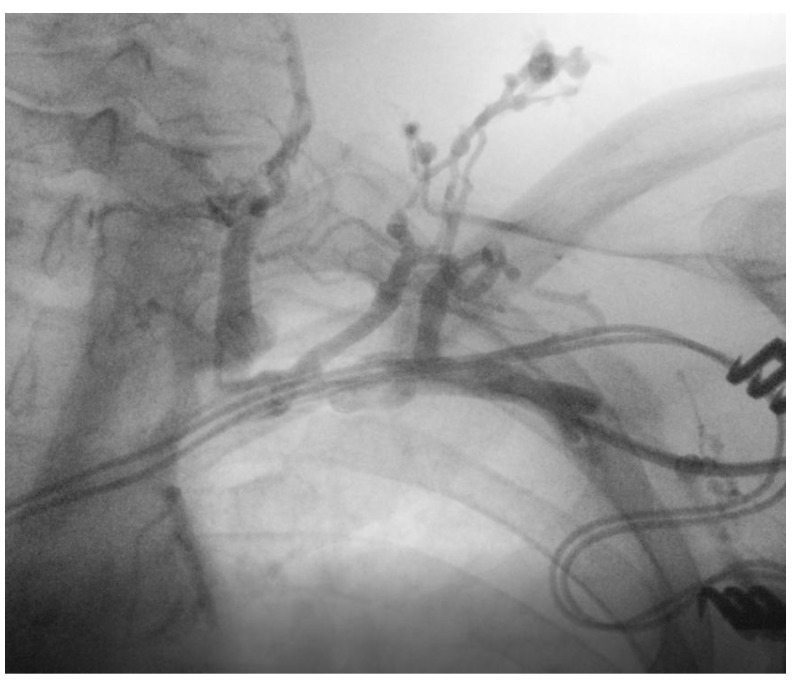
Occlusion of the left subclavian vein, with collateral circulation.

Iodinated contrast media was injected into the peripheral vein of the left arm, revealing occlusion of the left subclavian vein, with collateral circulation developed in the chest and neck and the suspicion of minimal circulation present on the deep subclavian venous axis (as shown in Figure 5).

Axillary vein puncture was performed, and a J-tip guidewire was advanced only up to the subclavian vein, indicating possible occlusion at this level. An Abbott 0.014′′ hydrophilic guide was introduced to identify the remaining lumen. The hydrophilic guide also stopped at this level, demonstrating the impossibility of passage on the deep venous axis due to occlusion.

As the patient was pacemaker-dependent, temporary cardiac pacing was performed using the right femoral vein approach, maintaining a stand-by heart rate of 50 beats/min, and hemodynamically monitoring was assured. 

After deep analgesia-sedation, an incision was made, and the pulse generator was extracted. The two leads were disconnected and dissected up to the sutures at the level of the cephalic vein, to release them from the scar tissue. Tie-down sutures were removed, and gentle traction was made to easily mobilize the two leads, without the possibility of withdrawing them. 

At that time, it was decided to extract the RV (dwell time 7 years) lead, being the only possibility to reach into the heart and to replace it with a single-coil defibrillation lead. 

The same technique described in Case 1 was used: a Liberator locking stylet was advanced up to the top of the lead, locked at this level, and secured using a one-tie accessory. The right atrium (RA) lead was secured with a guidewire. With continuous traction, a 9 French bidirectional, rotational, mechanical lead-extraction sheath (Evolution RL, Cook Medical, Bloomington, IN, USA) was advanced with difficulty over the lead, up to the left brachiocephalic vein, without the possibility of going further (as shown in Supplementary Video S3). Under these circumstances, the sheath was withdrawn, revealing large deposits of fibrin on the tip of the sheath. A new 11 French bidirectional, rotational, mechanical lead-extraction sheath (Evolution RL, Cook Medical, USA) was used and advanced over the lead up to the RA level. At this level, the RV lead detached from the RV apex was easily extracted (as shown in Supplementary Video S4).

**Figure 6:**
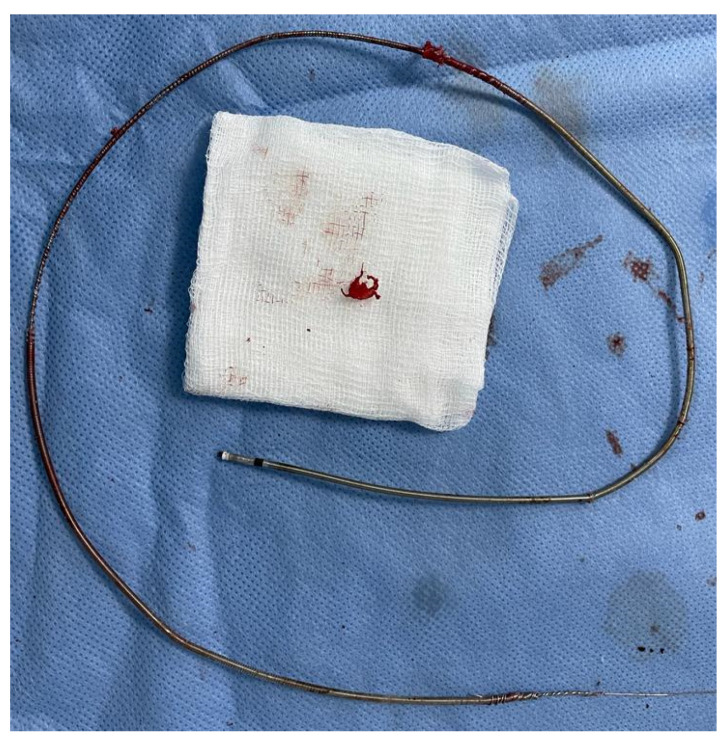
Extracted ventricular lead with large deposits of fibrin.

The extracted lead with the large deposits of fibrin removed from the tip of the 9 French bidirectional, rotational, mechanical lead-extraction sheath can be seen in Figure 6.

The outer sheath of the Evolution RL system was kept at the RA level. Two 0.035′′/180cm J-tip guidewires were inserted in the RA through this sheath. Afterward, the RA lead was checked, proving to have impedance, pacing, and sensing parameters within normal limits, which demonstrated that it was functional and could be preserved.

One of the 0.035′′/180 cm J-tip guidewires was used to introduce a Biotronik Selectra Bio 2 45 cm sheath (Biotronik, Berlin, Germany), and a single-coil, DF-4 active fixation defibrillation lead was inserted in the apical RV apex through this sheath. On the other remaining 0.035′′/180 cm J-tip guidewire, a Biotronik Selectra “Multipurpose EP (MPEP)” 55 cm sheath was used to cannulate the coronary sinus. After standard occlusive coronary sinus venography was performed, a suitable posterolateral vein was identified, and an IS4, OTW lead, was placed on that vein. After revision of the pre-pectoral pocket, the three leads were connected to a CRT-D.

The overall fluoroscopy time for this procedure was 15 min.

**Figure 7:**
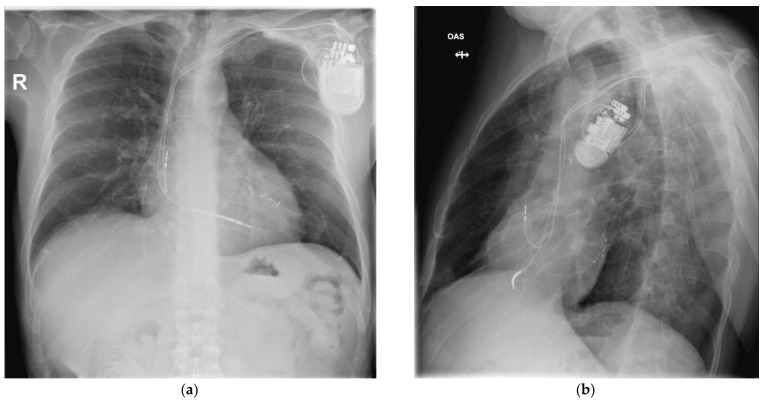
CRT-D system with leads in normal positions: (**a**) posteroanterior view and (**b**) left anterior oblique view.

Post-procedural echocardiography revealed no pericardial effusion, no additional tricuspid valve dysfunction, and no lead fragments. The control chest X-ray after the procedure revealed the leads were in normal positions, as shown in Figure 7.

### 2.3. Third Clinical Case

#### 2.3.1. Patient’s Medical History and Presentation

A 37-year-old female patient, with a cardiac resynchronization therapy pacemaker (CRT-P), was admitted to the Cardiology Department for the extraction of magnetic resonance non-conditional leads and implantation of a new magnetic resonance (MR)-conditional CRT-P.

The patient is known to have had a surgical correction of atrial and ventricular septal defects at 18 years. Right after the surgery, she developed a complete AV block, and a pacemaker with single unipolar with a passive fixation lead (non-MR-conditional) was implanted on the right ventricle apex. At the age of 26, the pacemaker had been upgraded to a dual chamber pacemaker with the implantation of a bipolar active fixation lead (non-MR-conditional) in the right atrial appendage. Seven years after the upgrade to a dual chamber pacemaker, she was scheduled for a pacemaker replacement as the battery reached its elective replacement indicators (ERI). Since the patient complained about shortness of breath and fatigue at a moderate physical effort, and the ECG revealed a broad QRS complex with a left bundle branch block (LBBB)-like aspect, the decision to upgrade the system to CRT-P was made. A bipolar OTW (MR-conditional) was placed in the posterolateral vein. In 2020, she presented an epileptic seizure, a left frontal tumor was diagnosed on computed tomography, and MRI was recommended for a better evaluation and to establish the best neurosurgical approach.

**Figure 8:**
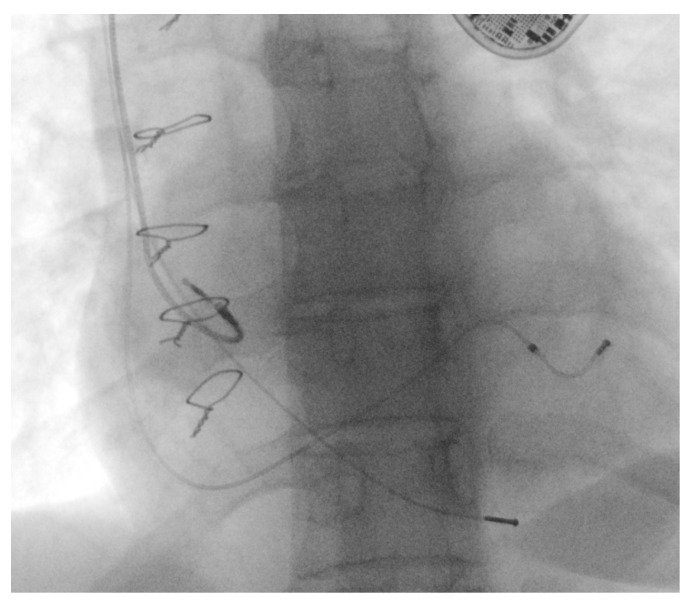
Chest X-ray at the time of admission at the hospital, with one lead positioned in the right atrial appendage, one on the apex of the right ventricle, and another one in the posterolateral vein.

At the time of admission to our department, the patient was asymptomatic. The ECG showed a sinus rhythm with biventricular paced activity triggered by the atrial activity. An echocardiogram revealed normal left ventricle function with preserved ejection fraction, mild tricuspid regurgitation, and no visible vegetations. A chest X-ray revealed a normal cardiothoracic ratio, a pulse generator PM placed subcutaneously in the left subclavian area, and three leads (as shown in Figure 8). 

Pacemaker interrogation revealed 100% ventricular paced and no underlying ventricular rhythm, with all leads having impedance, pacing, and sensing parameters within normal limits.

#### 2.3.2. Transvenous Leads Extraction Procedure

Temporary cardiac pacing was performed by the right femoral vein approach, maintaining a stand-by heart rate of 50 beats/min, as the patient was pacemaker-dependent.

After deep analgesia-sedation, an incision was made, and the pulse generator was extracted. The three leads were disconnected and dissected up to the sutures to release them from the scar tissue. The unipolar, passive fixation RV lead was 20 years old; the RA bipolar, active fixation lead was 12 years old; and the LV bipolar, OTW lead was 5 years old. Gentle traction was made to mobilize the three leads, without the possibility of withdrawing them.

**Figure 9:**
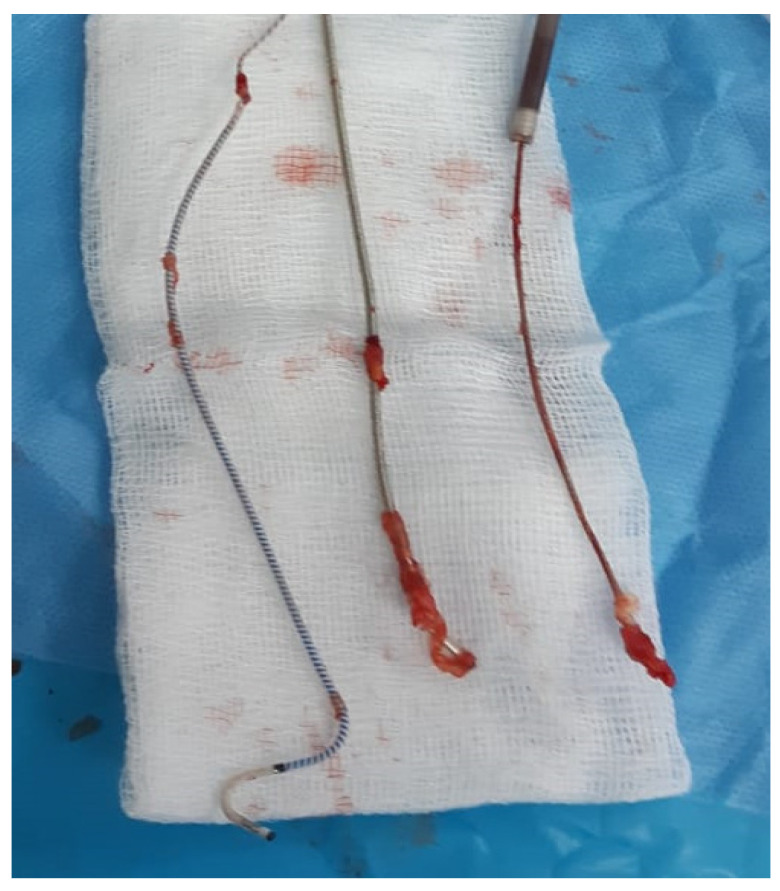
Extracted leads with excessive fibrous tissue on the distal end of RA and RV leads.

The connecter of the RA lead was cut off, and a Liberator locking stylet was advanced up to the top of the lead, locked at this level, and secured using a one-tie accessory. The RV and LV leads were secured with guidewires. With continuous traction, a 9 French bidirectional, rotational, mechanical lead-extraction sheath (Evolution RL, Cook Medical, USA) was advanced over the lead, up to the left innominate vein, where excessive fibrosis surrounded all three leads. The extraction sheath was advanced with difficulty, and the lead was extracted (as shown in Supplementary Video S5). The RV and LV leads were extracted using the same technique (Supplementary Video S6 and Supplementary Video S7, respectively). After removing the RV lead, the outer sheath was kept at the RA level. One 0.035′′/180 cm J-tip guidewire was inserted in the RA through the outer sheath, being used to introduce the other two 0.038′′/50 cm J-tip guidewires. Figure 9 shows the extracted leads with excessive fibrous tissue on the distal end of the RA and RV leads.

The 0.035′′/180 cm J-tip guidewire was used to introduce a LV OTW BP lead through Biotronik Selectra Bio 2 45 cm sheath (Biotronik, Germany) in the posterolateral vein. The impedance, pacing, and sensing parameters were within normal limits, and the patient did not have phrenic stimulation at high voltage. The other guidewires were used to introduce an RV bipolar, active fixation lead on the apex of the RV and an RA bipolar active fixation lead on the right atrial appendage. All three leads were connected to a CRT pacemaker placed on the revised left pre-pectoral pocket. All three leads and the pacemaker are MRI compatible.

During the TLE procedure, the patient remained hemodynamically stable, and the echocardiography evaluation revealed no pericardial effusion, tricuspid valve lesions, or remaining lead fragments.

The overall fluoroscopy time for this procedure was 8 min.

One month later, the neurosurgical intervention was performed with complete macroscopic ablation of the left frontal tumor formation and gyral resection. Postoperative, periodic cerebral MRI investigations were required to monitor the patient’s status.

## 3. Discussion

Transvenous lead extraction, the gold standard for lead removal, is a procedure that may involve several complications, so the decision to perform it must carefully weigh the risks and benefits. In this paper, we present three clinical cases of patients who presented different indications of TLE and explain how the procedures were successfully performed.

### 3.1. TLE Organizations and Security Measures

Due to organizational problems and economic issues, TLE centers use a different approach in the application of safety requirements; in some centers, the TLE procedure is performed in an electrophysiology, catheterization, or laboratory room, with deep analgesia-sedation, while in other centers, TLE is performed in an operating room/hybrid room, with general anesthesia and additional monitoring of the procedure (transesophageal echocardiography and arterial line), in the presence of a cardiac surgeon [10,11]. Transesophageal echocardiography, when used as a monitoring tool during TLE, was observed to provide higher rates of complete procedural success and a reduced risk of major complications [12]. 

The location where TLE procedure is performed is less important than the immediate availability of cardiothoracic surgical intervention. The time to surgical intervention is the most important factor in preventing death due to a major complication; therefore, organizational conditions should ensure the possibility of performing an emergency sternotomy within 5–10 min from the onset of the first symptoms of major complication [13,14].

Nowadays, personalized medicine, adapted to the particular situation of each patient, is the correct attitude. There are complex cases (with a large number of abandoned leads, very old leads, elderly or frail patients, and multiple comorbidities). In these situations, it is safer to start the procedure under general anesthesia and with transesophageal echocardiographic guidance. There are also cases, with only one or two leads to be extracted, in which general anesthesia and transesophageal ultrasound are usually not necessary during the procedure, knowing that many leads, especially those with a few years of dwell time, can be extracted by free traction without needing extraction materials. However, it is true that during the TLE procedure, the interventional team must have the ability to quickly change the work strategy depending on the case’s evolution.

### 3.2. Particularities of the Cases

For the first clinical case, biochemical evaluation excluded systemic inflammation, and the blood cultures and wound-drainage cultures excluded local infection, but given the risk of underlying endocarditis, the pocket erosion associated with skin retraction was a clear indication for the total removal of the entire ICD system [15]. In this case, due to an abandoned lead in the tissues from many years ago, it was necessary to remove two dual-coil ICD leads. This complicated the procedure and extended the fluoroscopy time. Safe deactivation of the anti-tachycardia therapies of the ICD device was required before the procedure, since electrocautery was used close to the device. Electrocautery can cause electrical interference that may interfere with the function of implanted devices and, for patients with ICDs, it can even cause inappropriate internal electric shocks [16]. The extent and nature of the scar tissue structure in the CIED pocket walls was correlated with the relative position of cardiac lead loops concerning the device itself. Lead movements underneath the device can lead to pocket-wall irritation in the capsule-formation phase, resulting in more extensive scarring formation [17]. 

In the second case, the simple upgrade from a dual-chamber system to cardiac resynchronization therapy was initially desired, which would have involved only the introduction of a new lead for LV pacing. The impossibility of passing through the deep venous axis due to occlusion of the left subclavian vein during the procedure determined the decision to remove the RV lead and replace it with a single-coil defibrillation lead. In this way, all the guidelines’ recommendations for patients with ischemic cardiomyopathy were fulfilled, knowing that CRT-D has been associated with a significant reduction in the risk of all-cause mortality compared with CRT-P in these patients [9].

Studies reported that total occlusion of the subclavian or brachiocephalic vein can occur on average in 12% (range 2–22%) of patients without an infection and with normally functioning leads [18]. Moreover, several hypotheses support the idea that more leads within a vein may be associated with a higher rate of occlusion [18]. Thus, this complication is not uncommon after CIED implantation [19]. In this case, the fluoroscopy time was slightly longer due to the complexity of the procedure. The outer sheath of the extraction system was kept at the RA level and used as a path for the two guidewires. They were utilized to introduce the single-coil defibrillation lead in the RV and to cannulate the coronary sinus for the LV lead.

The main interaction between the MRI and the non-MR-conditional pacemaker lead occurs due to the radiofrequency (RF) field generated by an MRI machine. If exposed to an MRI field, it causes adverse effects such as inappropriate device function due to interaction with the magnetic reed switch, device reset, pacing or sensing problems, changes in sensing or capture thresholds, and even lead perforation due to heating at the lead tip [9]. 

The advancement of MR-conditional technology has led to more complex clinical issues and better options for patient management. The current MR-conditional pacing technology provides solutions to some specific issues related to MR scanning [20]. In the case of the third patient, with repeated upgrades over time, the CRT pacemaker worked very well. However, the diagnosis of severe neurosurgical pathology required the replacement of the entire system with a new MRI-conditional one, to allow for a brain MRI examination and better management of the brain tumor. The TLE procedure was not without risks in a young patient who is completely pacemaker-dependent, but it was necessary and beneficial for MRI access, not only for the first evaluation before surgery but also for several periodic brain MRI investigations for follow-up.

It was reported that patients with a non-MRI-conditional CIED can perform an MRI at a field strength of 1.5 tesla without significant adverse events [21,22]. Even the current European Society of Cardiology guideline states that 1.5 tesla MRI scans (limited to SAR < 1.5 W/kg) may be considered in selected patients. The scans should be extrathoracic, and patients should not be pacemaker-dependent, taking into account the risk–benefit ratio of each patient [9]. For the patient in the third clinical case, a 3 tesla MRI brain examination was recommended for the evaluation of the tumor; since the patient was pacemaker-dependent, we decided to extract the leads and implant a new MR-conditional CRT-P, to avoid any risk during the MRI scanning or afterward.

Dual-coil leads are associated with more fibrosis and tissue in-growth, increasing the difficulty of extraction. The presence of two dual-coil implantable cardioverter-defibrillators causes significantly increased procedural risks [23].

Fibrotic scar tissue develops in areas where the leads are in contact with the endothelium. The first step is characterized by the thrombus formation along the lead at the time of implantation. The second step is characterized by the fibrosis of the thrombus resulting in almost complete encapsulation of the lead with a fibrin sheath, within 4–5 days of the implant procedure [24,25]. The venous entry site, the superior vena cava, and the electrode–endocardial interface are the most commons sites of adhesion formation [26]. In many patients, multiple areas of scar tissue are found, and this fibrotic tissue resists lead extraction, which may complicate the extraction procedure [27].

Increased precautions should be taken when scheduling patients with venous occlusion for TLE, as the lead extraction may be more difficult in these patients, requiring more advanced tools and more time. Older occlusions consisting of heavy fibrosis or even calcified tissue may require advanced extraction tools such as dilator sheaths and evolution sheaths [28]. 

Even if dual-coil leads are associated with greater procedural extraction risk due to fibrotic tissue in-growth into the proximal coil, another dual-coil lead was used in the first clinical case, since it provides superior defibrillation for a right-sided implant [29].

During extraction, uncoiling of the leads can happen, which results in retained components or failed extraction. To prevent this, a locking stylet was used to pull the lead from the tip and seal the tip of the lead during extraction. A one-tie compression coil was used for all procedures to aid in the removal of the lead, by binding the proximal components together and to the engaged locking stylet [23].

### 3.3. Advantages of Rotational, Mechanical Extraction Devices

The first generation of rotational, mechanical extraction devices had a unidirectional rotation mechanism, which was found to cause a phenomenon known as ‘lead wrapping’ in the presence of companion leads. Thus, the second generation of rotational, mechanical extraction devices have been designed to address this issue. The bidirectional, rotational mechanism seems to have a less aggressive tip, reducing the risk of damage to vascular structures, leads, or myocardial tissue [30].

Very commonly used bidirectional, rotational mechanism sheaths are the Evolution mechanical dilator sheath and TightRail™ mechanical dilator sheath. 

The Evolution mechanical dilator sheath (Cook Medical, Bloomington, IN, USA) uses a rotational mechanism with a stainless-steel bladed tip to overcome fibrosis and cut adherences. The outer sheath covers the cutting edge when cutting activity is not desired, so venous walls are protected from damage. In addition, a shorter Evolution dilator sheath (Shortie) has been designed with a sharper blade to facilitate venous access in cases with extensive calcification under the clavicle [31]. 

The TightRail™ mechanical dilator sheath (Spectranetics Corp., Colorado Springs, CO, USA) has a more flexible shaft with a shielded metal blade at the distal tip. The flexible shaft facilitates progression through tortuous vascular structures and fibrotic and calcific attachments. Moreover, the dilating metal blade at the distal tip is shielded until activated [32].

Results from a multicenter Italian registry that enrolled 124 patients with 238 lead extracts reported a clinical success rate of 100%, with no deaths or major complications and only five minor complications (4%), when an Evolution R bidirectional, rotational mechanical lead-extraction sheath was used [30]. Other studies that evaluated the TightRail ™ reported the same kind of results. Choi et al. successfully and safely used a TightRail ™ Evolution R bidirectional, rotational, mechanical lead-extraction sheath to extract 131 leads from 86 patients, whose longest lead age was less than 10 years [33]. A more recent study by Mazzone et al. reported the safety and efficacy of the bidirectional, rotational, mechanical sheath TightRail™ for 57 lead extractions in 26 patients [34].

When comparing the two types of rotational, mechanical dilator sheaths regarding the efficacy and safety of transvenous lead extraction in different clinical indications for 163 lead extractions in 98 consecutive patients, the conclusion was that using the Evolution and the TightRail rotational, mechanical dilator sheaths was similarly effective and safe [35].

For all three cases presented, the fluoroscopy time was reduced. These bidirectional powered-extraction sheaths with rotating sharp blades at the tip are a safe method that allows for both fluoroscopy and the overall procedural time to be reduced and ensures better patient comfort [35].

### 3.4. Practical Approaches When Performing TLE Using Rotational, Mechanical Extraction Devices

In order to reduce the risk of complications during TLE procedures, we suggest paying special attention to the following steps:

- a contrast dye injection should be performed on the peripheral vein of the involved region to highlight the deep venous and collateral circulation;
- it is essential to insert the locking guide (locking-stylet type) up to the tip of the lead in order to stiffen the lead as much as possible;
- it is essential to always perform a light traction of the assembly that indicates use in the fixation areas: subclavian vein, left brachiocephalic vein or only at the tip of the lead in RA and RV;
- it is essential to choose the extraction sheath well, usually 2 French larger than the lead’s diameter;
- it is essential to exert a continuous and constant traction force to achieve an optimal rotational sheath’s rail, without rash tractions;
- in certain situations, it is profitable to use a short sheath and to release the lead at the level of the subclavian vein and the brachiocephalic vein, which should then be replaced with a long sheath in order to release the lead up to its tip;
- the use of a counterattraction sheath, handled by a second operator, might be useful to fix the fibrin ties and to facilitate their cutting as well as to prevent inversion of the ventricular wall at the lead’s tip and to facilitate its release.

## 4. Conclusions

Transvenous lead extraction is regarded as the first-line strategy for the management of complications associated with cardiac implantable electronic devices when lead removal is indicated. The decision to perform a lead extraction should take into consideration not only the clinical indication for the procedure but also many other factors such as risks versus benefits, extractor and team experience, and even patient preference. Bidirectional, rotational mechanism sheaths can be safely used for these procedures.

## Supplementary Materials

The following are available online at https://www.mdpi.com/article/10.3390/ijerph20010379/s1.
